# Adult-Onset Gilles de la Tourette Syndrome: Psychogenic or Organic? The Challenge of Abnormal Neurophysiological Findings

**DOI:** 10.3389/fneur.2019.00461

**Published:** 2019-05-03

**Authors:** Viviana Versace, Stefania Campostrini, Luca Sebastianelli, Mirco Soda, Leopold Saltuari, Sigrid Lun, Raffaele Nardone, Markus Kofler

**Affiliations:** ^1^Department of Neurorehabilitation, Hospital of Vipiteno, Vipiteno, Italy; ^2^Research Unit for Neurorehabilitation South Tyrol, Bolzano, Italy; ^3^Department of Neuropsychology, Hospital of Bressanone, Bressanone, Italy; ^4^Department of Neurology, State Hospital Hochzirl, Zirl, Austria; ^5^Department of Psychiatry, Hospital of Bressanone, Bressanone, Italy; ^6^Department of Neurology, Franz Tappeiner Hospital, Merano, Italy; ^7^Department of Neurology, Christian Doppler Medical Center, Paracelsus Private Medical University of Salzburg, Salzburg, Austria

**Keywords:** tics, gille de la tourette syndrome, blink reflex excitability recovery, blink reflex prepulse inhibition, psychogenic

## Abstract

Gilles de la Tourette syndrome (GTS) is characterized by multiple motor and vocal tics. Adult-onset cases are rare and may be due to “reactivation” of childhood tics, or secondary to psychiatric or genetic diseases, or due to central nervous system lesions of different etiologies. Late-onset psychogenic motor/vocal tics resembling GTS have been described. Neurophysiology may serve to differentiate organic from functional GTS. Altered blink reflex pre-pulse inhibition (BR-PPI), blink reflex excitability recovery (BR-ERC), and short-interval intracortical inhibition (SICI) have been described in GTS. We report a 48-years-old male, who developed numerous motor/vocal tics 2 months after sustaining non-commotional craniofacial trauma in a car accident. Both his father and brother had died earlier in car crashes. He presented with blepharospasm-like forced lid closure, forceful lip pursing, noisy suction movements, and deep moaning sounds, occurring in variable combinations, without warning symptoms or internal “urge.” Tics showed low distractibility and these increased with attention. Standard magnetic resonance imaging, electroencephalography, and evoked potentials were unremarkable. Neuropsychology diagnosed moderately impaired intellect, attention, and executive functions. Psychiatric assessment revealed somatization disorder and generalized anxiety. BR-PPI was unremarkable, while BR-ERC was enhanced, even showing facilitation at short intervals. SICI was markedly reduced at 1 and 3 ms and intracortical facilitation (ICF) was enhanced at 10 ms. The patient fulfilled Fahn and Williams' diagnostic criteria for a psychogenic movement disorder. Neurophysiology, however, documented hyperexcitability of motor cortex and brainstem. We suggest that—similar to what has been reported in psychogenic dystonia—a pre-existing predisposition may have led to the functional hyperkinetic disorder in response to severe psychic stress.

## Introduction

Gilles de la Tourette syndrome (GTS), first reported in 1885 by the homonymous French physician (1), is a neurodevelopmental disorder characterized by multiple motor and vocal/phonic tics in variable combination, with typical copro- and echophenomena. The behavioral symptomatology is attributable to the spectrum of obsessive-compulsive or attention deficit and hyperactivity disorders. According to current criteria (fifth edition of the Diagnostic and Statistical Manual of Mental Disorders, DSM-5) (2), the onset of tics should occur before the age of 18 years, and their persistence, also with fluctuations, should be longer than 1 year; drug abuse and other medical causes must be excluded. Prevalence is about 1% worldwide. Etiopathology of GTS may be multifactorial, including genetic susceptibility, environmental influences, immunological, and hormonal factors. Evidence from neuropathology, neuropharmacology, structural, and functional neuroimaging, and neurophysiology support the hypothesis of dysfunctional cortico-striato-pallido-thalamo-cortical networks (3).

Few documented cases of idiopathic adult-onset GTS have been reported to date, some of which were referred to “reactivation” of childhood tics (4–6). Mejia and Jankovic described a large cohort of secondary “tourettism,” also with tardive onset, in conjuction with other idiopathic hyperkinetic disorders, or secondary to ischemia/hemorrhage of the basal ganglia, brain injury, infectious diseases of the central nervous system, exposure to neuroleptic drugs, neuropsychiatric developmental disorders, psychiatric disorders, and genetic or chromosomal conditions (7). Somewhat more literature is available about late-onset psychogenic motor/vocal tics resembling GTS features (8–11).

Neurophysiological investigations have attempted to characterize neuropathophysiological alterations in GTS at the subcortical and cortical level. Pre-pulse inhibition of the blink reflex (BR-PPI), which is a neurophysiological sensorimotor gating phenomenon, is deficient in GTS (12), and in some psychiatric disorders [for a review Kohl et al. (13)]. The excitability recovery curve of the blink reflex (BR-ERC), a neurophysiological hallmark of brainstem interneuron excitability, is also altered in GTS (14).

Motor cortex hyperexcitability is also involved in GTS pathophysiology, as impaired intracortical inhibition has repeatedly been reported (15–18). Further evidence indicates cortical and brainstem synaptic plasticity abnormalities in GTS patients. Indeed, both intermittent and continuous theta burst stimulation failed to modulate the excitability of the primary motor cortex, and the brainstem circuits underlying the blink reflex remained unaffected by trains of facilitatory or inhibitory stimuli (19–21). In mild GTS, the application of a paired associative stimulation protocol (PAS) (22) failed to induce long-term potentiation (LTP)-like synaptic plasticity but rather induced an unexpected long-term depression (LTD)-like effect that inversely correlated to symptom severity (23). In contrast, Martin-Rodriguez et al. reported abnormally increased LTP-like motor plasticity after a PAS protocol, however only in severely affected GTS patients (24).

The dysfunction of inhibitory neural circuits at cortical, brainstem, and spinal level assessed through neurophysiological tests is considered diagnostic in movement disorders and often allows for differentiating organic from psychogenic forms. For example, the BR-ERC has been shown to be abnormal in essential but not in psychogenic blepharospasm (25).

An impaired short- and long-interval intracortical inhibition (SICI, LICI), is commonly considered to be a hallmark of several hyperkinetic disorders, but it was also detected in psychogenic dystonia (26–28). Therefore, although neurophysiological findings may provide evidence of abnormalities supporting a clinically challenging diagnosis, they may not always serve to unequivocally differentiate an organic from a functional movement disorder.

## Case Report

We report a 48-year-old male patient who was referred to our Neurological out-patient clinic due to a tic disorder resembling GTS.

One year prior, the patient had been involved in a car crash causing a non-commotional cranio-facial trauma. A cerebral computer tomography (CT) scan was unremarkable. However, the emotional impact of the accident on the patient was great, as he had lost his only brother a year before due to the sequalae of severe traumatic brain injury, which he had sustained in a major car accident. His father had also died earlier in a car crash. A month after the patient's accident, he began to develop involuntary stereotyped facial movements, such as forceful eye closure or grimacing with his mouth, as well as phonic tics such as pronouncing deep and prolonged sounds or vocalization. Their frequency of occurrence was high, with numerous attacks per day, but devoid of any particular triggering factor. Because of the socially disabling symptoms, he had to quit his job and isolated himself from his community.

Oral alprazolam (3 mg/d), sertraline (50 mg/d) and risperidone (2 mg/d), administered in sequence, were ineffective in mitigating the motor-vocal symptoms but they could make the patient feel “internally more quiet.”

Neurological examination was totally unremarkable except for intermittent motor tics such as blepharospasm-like forced lid closure, grimacing, forced lip closure, noisy suction movements and phonic tics like grunting, vocalizations (mostly a deep prolonged “ah”), not in a constant combination or sequence, and lasting several seconds. The tics increased with attention in intensity and frequency. At the end of an episode, the patient appeared mortified and apologized for the occurrence of the tics. On two occasions, attempts to distract the patient during a series of tics could interrupt a sequence of vocalizations. The patient, however, was unable to interrupt the tics voluntarily. He reported that the tics would arrive suddenly but denied a preceding internal urge or any warning signs.

The patient consented to videotaping (Supplement Videos S1, S2), and interestingly, during the recorded clinical examination, he presented more tics than during other times, e.g., while sitting seemingly unobserved in the waiting room.

The patient's history was unremarkable with respect to pre, peri-, and postnatal development. There was no family history of tics nor any other movement disorders. The family's socio-financial situation was difficult, and his parents could take care of him only marginally. He attended normal school until the age of 18 years, but with overall poor performance.

At the time of his first presentation at our institution, the patient lived with his elderly mother and had a part-time job without public contacts. He declared to be emotionally prostrated by the involuntary movements, and to be highly motivated to find treatment. The patient also complained of poor attention and memory, and that he feared of developing dementia. A two weeks trial with haloperidol (2 mg/d) failed to ameliorate the symptoms.

## Neuropsychological Examination

Overall, the patient presented with a globally reduced cognitive profile, when corrected for age and education. On the Wechsler Adult Intelligence Scale—Fourth Edition (WAIS-IV) (29), the patient obtained a Full Scale Intelligence Quotient (FSIQ) of 61, with demonstration of generally commensurate verbal and nonverbal intellectual abilities (Verbal Comprehension Index 71, Perceptual Reasoning Index 67, Working Memory Index 75, and Processing Speed Index 70). The comprehensive neuropsychological evaluation highlights a discontinuous profile characterized by multi-domain difficulties, in particular concerning attentional processes (prolonged reaction time, unstable processing velocity, fluctuating focused attention, distractibility, impairment of divided attention) and executive functions (reduced working memory, impaired verbal and visuo-spatial logical-abstract reasoning, reduced divergent thinking). Memory skills, semantic verbal fluency and visual-constructive abilities were preserved.

Although a WAIS-IV FSIQ score of 61 typically reflects moderately impaired intellectual functioning, given what emerged from the social anamnesis, the neuropsychological tests and the observed adaptive functioning, the patient's profile fitted with a borderline intellectual functioning.

## Psychiatric Assessment

On psychiatric evaluation, the patient met DSM-5 criteria for somatic symptom disorder, major depression, and generalized anxiety disorder, as well as personality features related to all three clusters A-C. The Millon Clinical Multiaxial Inventory, Third Edition (MCMI-III) showed slightly abnormal scores for social avoidance, paranoid borderline and passive-aggressive traits. Clinical exploration also documented low coping strategies, concurring with the patient's low intellectual profile, rendering malingering an unlikely relevant factor for the patient's tic disorder.

## Other Examinations

A 1.5 T magnetic resonance imaging (MRI) of the brain, as well as standard electroencephalography and somatosensory evoked potentials, failed to show any pathological findings. Blood and urine tests were unremarkable.

## Neurophysiologic Examinations

Due to the non-univocal diagnostic classification of the patient's symptoms and in order to rule out neuronal dysfunction despite normal imaging studies, several neurophysiological examinations were carried out in this patient so as to investigate excitatory and inhibitory trigemino-facial circuits within the brainstem and to assess inhibitory circuits of the primary motor cortex.

## Blink Reflex (BR)

The BR is a trigemino-facial reflex, which can be utilized neurophysiologically to assess excitability and conductivity of underlying brainstem circuitry (30).

After obtaining written informed consent from the patient, we performed blink reflex studies using routine electrodiagnostic equipment (Viking EDX System, Natus, Middleton, WI, USA). The patient was tested in the supine position with eyes open and looking straight ahead.

Single sweeps of electromyographic (EMG) activity of the orbicularis oculi muscle were recorded bilaterally with self-adhesive disposable electrodes attached to the skin, the active electrode overlying the middle portion of the muscle below each eye, and the reference electrode lateral to the outer canthus. The EMG signal was amplified (x1000), band-pass filtered (30–3,000 Hz) and rectified. BRs were evoked by electrical stimuli (0.5 ms rectangular pulses) delivered to the right supraorbital nerve (SON) with a standard bar electrode, cathode over the supraorbital notch and anode 3 cm above along the course of the nerve on the forehead. We used 10 times sensory threshold (ST) intensity to elicit the BR in 8 trials with at least 10 s interval between two consecutive trials. ST was defined as the minimum intensity perceived in at least 4 out of 8 trials. Latency and amplitude of the ipsilateral R1 component, as well as latency and area-under-the-curve (henceforth “area”) of the ipsilateral R2 and contralateral R2c components were measured in single traces and the values averaged.

The patient's ST for SON stimulation and all BR parameters (R1 latency and amplitude, R2 latency and area, R2c latency and area) were within the normal limits (31), see Table 1.

**Table 1:** Results of blink reflex (BR) and blink reflex modulation (BR-PPI, BR-ERC) studies.

		**Patient**	Normal values^*^
**BR (*****average of 8 trials)***			
ST for right SON stimulation	[mA]	1.3	1.9 ± 0.4
R1 latency	[ms]	10.6	10.9 ± 1.0
R1 amplitude	[μV]	89.3	196.4 ± 172.0
R2 latency	[ms]	33.6	33.0 ± 3.8
R2 area	[μV^*^ms]	2143	4186.2 ± 3030.9
R2c latency	[ms]	34.6	34.6 ± 3.9
R2c area	[μV^*^ms]	2097	2990.0 ± 1958.5
**BR-PPI** ***(mean of 8 test trials / mean of 8 control trials)***			
ST for right D2 stimulation	[mA]	2.6	1.9 ± 0.7
R1 amplitude	[%]	354.2	155.2 ± 101.2
R2 area	[%]	37.2	34.7 ± 15.1
R2c area	[%]	43.2	28.4 ± 16.9
**BR-ERC for R2 area** ***(mean of 4 conditioned / mean of 4 control trials for each ISI)***			
ISI 160 ms	[%]	120	20.4 ± 16.3
ISI 300 ms	[%]	94	39.9 ± 19.0
ISI 500 ms	[%]	82	62.4 ± 19.8
ISI 1000 ms	[%]	124	//

ST, sensory threshold; SON, supraorital nerve; D2, index finger; BR, blink reflex; BR-PPI, prepulse inhibition of BR; BR-ERC, excitability recovery curve of BR; ISI, interstimulus interval.

* *normal values for male subjects from Kofler et al. (31)*.

## Prepulse Inhibition of the Blink Reflex (BR-PPI)

BR-PPI is a neurophysiological measure of sensorimotor gating, testing inhibitory circuitry within the brainstem and its suprasegmental control. The amount of gating is reflected by the degree to which the reflex response is suppressed by a weak prepulse. PPI may occur with subthreshold stimuli of the same or another modality applied at appropriate interstimulus intervals (32). In healthy subjects, a prepulse modulation of the blink reflex produced a facilitation of the R1 component with ISIs from 25 to 60 ms and a suppression of the R2 and R2c components (“prepulse inhibition” per definition) with ISIs from 60 m to 125 ms (33).

In the present patient, SON stimuli were the same as described above for BR testing. Electrical stimuli used as a prepulse were delivered with ring electrodes attached to the patient's right index finger, using constant current square wave electrical stimuli of 0.2 ms duration at 2 ST intensity. Prepulse stimuli to the digital nerves were applied 100 ms before SON stimulation. Eight single sweeps were recorded with at least 10 s interval between two consecutive trials. BR parameters were measured in single traces and the values then averaged.

For analysis of prepulse effects, we measured R1 amplitude and R2/R2c area in each single rectified trace. PPI size was determined as the amount of suppression of the area of R2 and R2c, induced by index finger prepulses, expressed as percentages of the respective control trials (SON stimulation alone). Thus, smaller values represent more inhibition.

The patient's ST for index finger stimulation was 2.6 mA. Prepulses delivered to the right index finger caused a robust facilitation of the R1 amplitude (with the sporadic occurrence of a small contralateral R1 response, possibly due to volume conduction), and a normal PPI of the R2 and R2c components, in line with published normal values (31); see Table 1.

## Excitability Recovery Curve of the Blink Reflex (BR-ERC)

BR excitability can be tested by paired-pulse stimulation (conditioning and test stimuli) at varying ISIs to construct the BR-ERC (34, 35). In healthy subjects, paired-pulse stimulation of the SON leads to a significant decrease in the conditioned R2 area as compared to unconditioned R2 area up to 3 seconds (25). R2 is usually nearly completely abolished at ISIs from 0 to 300 ms, to slowly recover to reach about 30–50% at the 500 ms ISI, 40–80% at the 1,000 ms ISI, and 70–90% at the 1,500 ms ISI (30, 36).

In the present patient, for establishing BR-ERC of the R2 and R2c components, paired pulses of the same intensity were delivered to the right SON at the following ISIs: 160, 300, 500, and 1,000 ms (36, 37). Four traces were recorded and on-line averaged for each ISI with at least a 15 s pause between each stimulus pair.

For analysis of BR excitability recovery, we measured the area of R2 and R2c in in each single rectified trace and then calculated the ratio of conditioned divided by unconditioned response for both R2 and R2c separately, yielding percentage recovery for each ISI tested.

**Figure 1:**
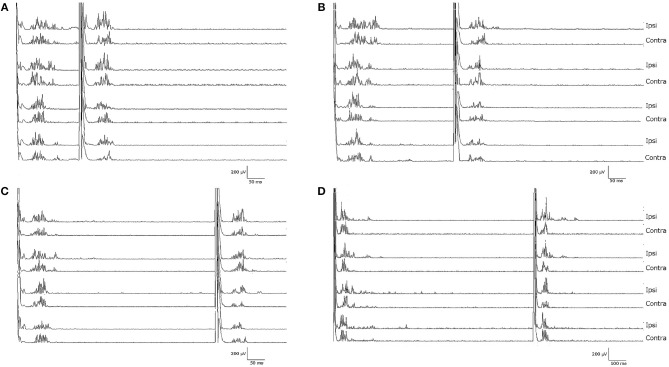
Blink reflexes of the patient following paired-pulse stimulation with different interstimulus intervals: **(A)** 160 ms, **(B)** 300 ms, **(C)** 500 ms, **(D)** 1,000 ms. Note the lack of inhibition of the R2 component of the conditioned blink reflex to the second stimulus, normally induced by the first test stimulus.

Notably, the patient presented with an almost complete loss of inhibition of R2 and R2c. At ISIs 160 and 1,000 ms, the conditioned R2 responses were even facilitated, while at ISIs 300 and 500 ms, the conditioned R2 responses were barely inhibited (see Figure 1 and Table 1). The contralateral R2c area showed a similar pattern of modulation.

## Transcranial Magnetic Stimulation—Short-Latency Intracortical Inhibition and Facilitation (SICI, ICF)

Transcranial magnetic stimulation (TMS) is a widely used technique to examine motor cortical physiology in humans. TMS is able to test inhibitory and facilitatory circuits within the motor cortex by using different stimulation paradigms (38). With a subthreshold conditioning stimulus followed by a suprathreshold test stimulus at ISIs of 1–6 ms, the motor evoked potential generated by the test stimulus is suppressed, which is known as short-interval intracortical inhibition (SICI). Conversely, motor evoked potentials are facilitated at ISIs 10–30 ms, which is termed as intracortical facilitation (ICF) (39).

We tested the patient and five age-matched healthy control subjects (4 males, 1 female, mean age 45 ± 2 years), after obtaining written informed consent. Surface EMG was recorded from the right first dorsal interosseous muscle at rest, while the participants sat in a comfortable reclining chair. TMS was applied to the contralateral motor cortex with a figure-of-eight coil connected to a Magstim Bistim 200^2^ stimulator (Magstim, Whitland, Dyfed, UK). The coil was placed in the optimal position for activation of the target muscle with induced current in the brain in the posterior-anterior direction. Resting motor threshold and TMS intensity necessary to elicit a motor evoked potential of 1 mV amplitude (test stimulus intensity) were determined (40). The patient's resting motor threshold was 49% of the maximum stimulator output. We tested the following ISIs: for SICI 1 and 3 ms, and for ICF 10 and 15 ms, in randomized order. Four stimuli were delivered for each ISI. Conditioning stimulus intensity was set at 80% of resting motor threshold (41). EMG responses were recorded using routine electrodiagnostic equipment (Viking EDX System, Natus, Middleton,WI, USA).

**Figure 2:**
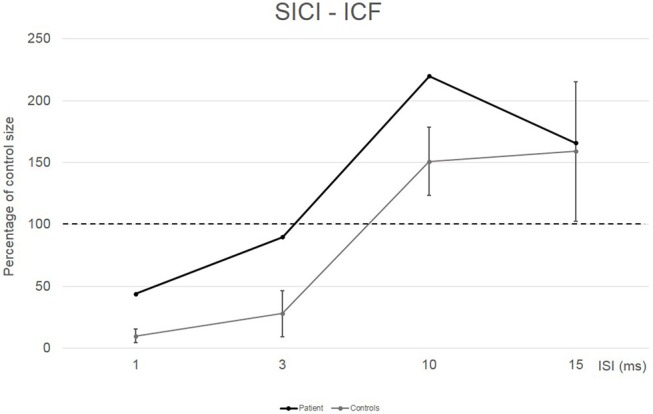
EMG responses in relaxed first dorsal interosseus muscle to paired-pulse transcranial magnetic stimulation at different interstimuslus intervals (ISIs) in the patient (black line) and in five controls (gray line). At each ISI, the size of the conditioned response is expressed as a percentage of the size of the control response (to test stimulus alone).

Conditioned MEP amplitudes for each ISI are expressed as a percentage of the size of control MEPs. The patient showed a reduced amount of SICI at ISI 1 ms (44%) and a completely absent SICI at ISI 3 ms (90%) compared to the control group (10% for ISI 1 ms and 28% for ISI 3 ms). Patient's ICF amount was larger than in the control group at ISI 10 ms (220 vs. 151%) (see Figure 2).

## Discussion

We report a patient with borderline intellectual functioning who developed facial motor and phonic tics shortly after a car accident causing a non-commotional head injury.

A diagnosis of functional motor-phonic GTS-like tics was established by neurologists trained in movement disorders (VV, LSe, LSa, RN, MK), together with a neuropsychologist (MS) and a psychiatrist (SL), after a comprehensive diagnostic work-up on the following basis: adult tic-onset shortly after a major psychic stress, borderline intellectual functioning (which may have led to somatization based on limited mental resources to cope with a major psychic trauma), incongruence and inconsistency of clinical signs and symptoms (lack of premonitory sensations and of internal urge, inability to suppress the movements, lack of rebound phenomena, augmentation of tics while being observed or filmed, with prolonged and accentuated presentation of the entire repertoire of tics), absence of coprophenomena, some distractibility on few occasions, lack of clinical improvement upon intake of neuroleptics or benzodiazepines, and—ultimately—a psychiatric diagnosis of somatoform disorder and generalized anxiety. Thus, the patient fulfilled the Fahn and Williams' diagnostic criteria for clinically established psychogenic movement disorder (42).

Moreover, all routine examinations (cerebral CT and MRI; EEG, evoked potentials, blood tests) were unremarkable, excluding secondary tourettism due to, e.g., basal ganglia lesion after head trauma.

Further electrophysiological tests, however, revealed hyperexcitability of the primary motor cortex and of brainstem circuits mediating the BR. Impaired SICI has been reported in both organic and functional dystonic patients (26, 28, 43) and in GTS (15–18). However, an abnormally enhanced BR-ERC has been reported in GTS and has allowed distinguishing essential from psychogenic blepharospasm (25). Espay et al. (26) observed similarly abnormal neurophysiological findings (reduced SICI, LICI, and cortical silent period; increased cutaneous silent period) in both organic and functional dystonia. Based on their findings, they proposed that abnormal cortical and spinal excitability in the central nervous system in organic dystonia may, in part, be a consequence rather than a cause of abnormally prolonged and altered movements and postures in dystonia. As psychogenic and organic dystonia share similar neurophysiological abnormalities, they proposed as an alternative explanation that their findings may represent endophenotypic abnormalities that may predispose to either type of dystonia (26).

In analogy, we propose that in the present patient both reduced SICI as well as disinhibited trigemino-facial reflexes, which are a neurophysiological feature of “true” GTS, may reflect a pre-existing idiopathic condition that may have caused the development of a hyperkinetic disorder in the course of his life. Several elements link this patient's characteristics to those typical of functional movement disorders, but also to those characteristic of organic GTS. Is there a gray overlap zone between patients with neurological conversion disorders and patients with organic neurological disorders?

Functional imaging techniques and neurophysiology have provided unequivocal evidence that conversion disorders have indeed a neurobiological basis but are often triggered by psychic factors [for a review: (44, 45)].

Historically, functional movement disorders were assumed to be based on psychological causation. The expectation, however, that psychological trauma, either at time of onset of the physical symptoms or earlier, would be causal to psychogenic disorders was not always fulfilled. This discrepancy led to interest in a neurobiologically informed model of hierarchical Bayesian inference in the brain, explaining functional motor and sensory symptoms in terms of perception, and action arising from inference based on prior beliefs and sensory information (46). Following this model, primary or secondary failure of inference may explain functional disorders. “Primary failure” describes the autonomous emergence of a strong percept or belief (following top-down attentional modulation of synaptic gain) (46). “Secondary failure” refers to the false interpretation of the ensuing percept as a symptom explaining the lack of predictability of its content by the source of attentional modulation. This model accommodates a number of fundamental observations of functional motor and sensory symptoms, e.g., (i) their induction and maintenance through attention, (ii) their modification through expectation, prior experience, and cultural beliefs; and (iii) their involuntary and symptomatic nature (46). Patients report movements in functional movement disorders as being involuntary and incontrollable, although they demonstrate characteristics of voluntary movement such as distractibility, improvement with placebo, and the presence of pre-movement potentials. This reasoning led to the assumption of a brain mechanism which allows for the occurrence of voluntary movement that is subjectively interpreted as involuntary.

Cognitive neuroscience does demonstrate that mechanisms in the brain exist which provide sense of intention and of agency to movement, which can be selectively disrupted (47). The temporoparietal junction could be identified as an area which compares actual with predicted sensory feedback. The feeling of involuntary movement might be produced through hypoactivity in that region, representing failure in matching actual and predicted sensory feedback (48). Additionally, emotionally arousing events might trigger movement in functional movement disorders as controlled by the supplementary motor area that is functionally disconnected from top-down control by the prefrontal cortex (49, 50).

Overrating the number of tremors per day in patients with functional tremor vs. those with organic tremor is a discrepancy which has been interpreted within this Bayesian framework as a dominance of prior expectancy over sensory data (51).

The importance of psychological factors (anxiety, depression, arousal, and attention) should not be ignored, of course. However, it should be kept in mind that psychological trauma is not necessarily the only cause of the presence of a functional disorders. The common occurrence of physical triggering events, such as illness or injury in patients with pure functional symptoms, in fact emphasizes the overlap between organic and non-organic illness (52, 53).

The present patient did indeed develop a somatization disorder following psychic stress; the phenotypic manifestation presented as facial motor tics and vocal-phonic tics. Among abnormal movements, which represent the most frequent symptoms of psychogenic neurological disorders, tics account for about 8%; the most prevalent body parts involved in movement phenomenology are face and lips (54).

In summary, our patient presents with a puzzling mixture of clinical signs of functional movement disorder, supported by a variety of normal laboratory findings, but at the same time neurophysiological evidence of abnormal excitability at the cortical and brainstem level. In accordance with Espay et al. (26), we propose that neurophysiological abnormalities may represent an epiphenomenon of an endophenotypic predisposition for organic or psychogenic hyperkinetic syndromes in certain patients.

## Informed Consent

A written informed consent was obtained from the patient for the publication of this case report.

## Data Availability

All datasets generated for this study are included in the manuscript and/or the Supplementary Files.

## Ethics Statement

The local ethics committee approval is not required for clinical routine diagnostic examination.

## Author Contributions

VV, SC, MK, MS, LSe, and SL performed the acquisition of data, the drafting/revising of the manuscript and accepted responsibility for conduct of research and final approval. LSa, MK, and RN performed the drafting/revising of the manuscript and accepted responsibility for conduct of research and final approval.

### Conflict of Interest Statement

The authors declare that the research was conducted in the absence of any commercial or financial relationships that could be construed as a potential conflict of interest.

## Supplementary Material

Supplement Videos S1, S2 A usual sample of patient's tics.

## References

[1] de la Tourette GG Etude sur une affection nerveuse caracterisée par de l'incoordination motrice accompagnée d'echolalie et de coprolalie. Arch Neurol. (1885) 9:19–42,158–200.

[2] American Psychiatric Association Diagnostic and Statistical Manual of Mental Disorders, 5th edn (DSM-5). Arlington: American Psychiatric Publishing (2013). 10.1176/appi.books.9780890425596

[3] Hallett M. Tourette syndrome: update. Brain Dev. (2015) 37:651–5. 10.1016/j.braindev.2014.11.005 25604739 PMC4475482

[4] Tijssen MA Brown P Morris HR Lees A. Late onset startle induced tics. J Neurol Neurosurg Psychiatry. (1999) 67:782–4. 10567498 10.1136/jnnp.67.6.782 PMC1736657

[5] Chouinard S Ford B. Adult onset tic disorders. J Neurol Neurosurg Psychiatry. (2000) 68:738–43. 10.1136/jnnp.68.6.738 10811697 PMC1736950

[6] Jankovic J Gelineau-Kattner R Davidson A. Tourette's syndrome in adults. Mov Disord. (2010) 25:2171–5. 10.1002/mds.23199 20690167

[7] Mejia NI Jankovic J. Secondary tics and tourettism. Rev Bras Psiquiatr. (2005) 27:11–7. 10.1590/S1516-44462005000100006 15867978

[8] Factor SA Podskalny GD Molho ES. Psychogenic movement disorders: frequency, clinical profile, and characteristics. J Neurol Neurosurg Psychiatry. (1995) 59:406–12. 7561921 10.1136/jnnp.59.4.406 PMC486078

[9] Frías A Sierra R Llorens P. Conversive Gilles de la Tourette syndrome. a case report. Actas Esp Psiquiatr. (2009) 37:115–7. 19401860

[10] Baizabal-Carvallo JF Jankovic J. The clinical features of psychogenic movement disorders resembling tics. J Neurol Neurosurg Psychiatry. (2014)85:573–5. 10.1136/jnnp-2013-305594 24259592

[11] Vale TC Pedroso JL Knobel M Knobel E. Late-onset psychogenic chronic phonic-tics. Tremor Other Hyperkinet Mov. (2016) 6:387. 10.7916/D88S4PWW 27375961 PMC4925920

[12] Castellanos FX Fine EJ Kaysen D Marsh WL Rapoport JL Hallett M. Sensorimotor gating in boys with Tourette's syndrome and ADHD: preliminary results. Biol Psychiatry. (1996) 39:33–41. 8719124 10.1016/0006-3223(95)00101-8

[13] Kohl S Heekeren K Klosterkötter J Kuhn J. Prepulse inhibition in psychiatric disorders–apart from schizophrenia. J Psychiatr Res. (2013) 47:445–52. 10.1016/j.jpsychires.2012.11.018 23287742

[14] Smith SJ Lees AJ. Abnormalities of the blink reflex in Gilles de la Tourette syndrome. J Neurol Neurosurg Psychiatry. (1989) 52:895–8. 2769283 10.1136/jnnp.52.7.895 PMC1031940

[15] Ziemann U Paulus W Rothenberger A. Decreased motor inhibition in Tourette's disorder: evidence from transcranial magnetic stimulation. Am J Psychiatry. (1997) 154:1277–84. 9286189 10.1176/ajp.154.9.1277

[16] Gilbert DL Bansal AS Sethuraman G Sallee FR Zhang J Lipps T. Association of cortical disinhibition with tic, ADHD, and OCD severity in Tourette syndrome. Mov Disord. (2004) 19:416–25. 10.1002/mds.20044 15077239

[17] Orth M Munchau A Rothwell JC. Corticospinal system excitability at rest is associated with tic severity in Tourette syndrome. Biol Psychiatry. (2008) 64:248–51. 10.1016/j.biopsych.2007.12.009 18243162

[18] Orth M Rothwell JC. Motor cortex excitability and comorbidity in Gilles de la Tourette syndrome. J Neurol Neurosurg Psychiatry. (2009) 80:29–34. 10.1136/jnnp.2008.149484 18931001

[19] Suppa A Belvisi D Bologna M Marsili L Berardelli I Moretti G Abnormal cortical and brainstem plasticity in Gilles de la Tourette syndrome. Mov Disord. (2011) 26:1703–10. 10.1002/mds.23706 21442662

[20] Wu SW Gilbert DL. Altered neurophysiologic response to intermittent theta burst stimulation in Tourette syndrome. Brain Stimul. (2012) 5:315–19. 10.1016/j.brs.2011.04.001 22037119

[21] Suppa A Marsili L Di Stasio F Berardelli I Roselli V Pasquini M. Cortical and brainstem plasticity in Tourette syndrome and obsessive-compulsive disorder. Mov Disord. (2014) 29:1523–31. 10.1002/mds.25960 24996148

[22] Stefan K Kunesch E Cohen LG Benecke R Classen J. Induction of plasticity in the human motor cortex by paired associative stimulation. Brain. (2000) 123:572–84. 10.1093/brain/123.3.572 10686179

[23] Brandt VC Niessen E Ganos C Kahl U Baumer T Munchau A. Altered synaptic plasticity in Tourette's syndrome and its relationship to motor skill learning. PLoS ONE. (2014) 9:e98417. 10.1371/journal.pone.0098417 24878665 PMC4039486

[24] Martín-Rodríguez JF Ruiz-Rodríguez MA Palomar FJ Cáceres-Redondo MT Vargas L Porcacchia P. Aberrant cortical associative plasticity associated with severe adult Tourette syndrome. Mov Disord. (2015) 30:431–5. 10.1002/mds.26151 25649686

[25] Schwingenschuh P Katschnig P Edwards MJ Teo JT Korlipara LV Rothwell JC. The blink reflex recovery cycle differs between essential and presumed psychogenic blepharospasm. Neurology. (2011) 76:610–4. 10.1212/WNL.0b013e31820c3074 21321334 PMC3053342

[26] Espay AJ Morgante F Purzner J Gunraj CA Lang AE Chen R. Cortical and spinal abnormalities in psychogenic dystonia. Ann Neurol. (2006) 59:825–34. 10.1002/ana.20837 16634038

[27] Avanzino L Martino D van de Warrenburg BP Schneider SA Abbruzzese G Defazio G. Cortical excitability is abnormal in patients with the “fixed dystonia” syndrome. Mov Disord. (2008) 23:646–52. 10.1002/mds.21801 18175341

[28] Quartarone A Rizzo V Terranova C Morgante F Schneider S Ibrahim N Abnormal sensorimotor plasticity in organic but not in psychogenic dystonia. Brain. (2009) 132(Pt 10):2871–7. 10.1093/brain/awp213 19690095 PMC2997979

[29] Wechsler D Wechsler Adult Intelligence Scale, 4th edn. San Antonio, TX: Pearson (2008).

[30] Valls-Solé J. Assessment of excitability in brainstem circuits mediating the blink reflex and the startle reaction. Clin Neurophysiol. (2012) 123:13–20. 10.1016/j.clinph.2011.04.029 22030138

[31] Kofler M Kumru H Schaller J Saltuari L. Blink reflex prepulse inhibition and excitability recovery: influence of age and sex. Clin Neurophysiol. (2013) 124:126–35. 10.1016/j.clinph.2012.07.001 22857876

[32] Valls-Solé J Valldeoriola F Molinuevo JL Cossu G Nobbe F. Prepulse modulation of the startle reaction and the blink reflex in normal human subjects. Exp Brain Res. (1999) 129:49–56. 10550502 10.1007/s002210050935

[33] Valls-Solé J Cammarota A Alvarez R Hallett M. Orbicularis oculi responses to stimulation of nerve afferents from upper and lower limbs in normal humans. Brain Res. (1994) 650:313–6. 7953697 10.1016/0006-8993(94)91797-3

[34] Kimura J Harada O. Recovery curves of the blink reflex during wakefulness and sleep. J Neurol. (1976) 213:189–98. 61259 10.1007/BF00312869

[35] Berardelli A Cruccu G Kimura J Ongerboer de Visser BW Valls-Sole J The orbicularis oculi reflexes. Recommendations for the practice of clinical neurophysiology. Electroencephalogr Clin Neurophysiol Suppl. (1999) 52:249–53. 10590992

[36] Kumru H Vidal J Kofler M Portell E Valls-Solé J Alterations in excitatory and inhibitory brainstem interneuronal circuits following severe spinal cord injury. J Neurotrauma. (2010) 27:721–8. 10.1089/neu.2009.1089 20067395

[37] Kimura J Harada O. Recovery curves of the blink reflex during wakefulness and sleep. J Neurol. (1976) 213:189–98. 61259 10.1007/BF00312869

[38] Ziemann U. Intracortical inhibition and facilitation in the conventional paired TMS paradigm. Electroencephalogr Clin Neurophysiol Suppl. (1999) 51:127–36. 10590943

[39] Kujirai T Caramia MD Rothwell JC Day BL Thompson PD Ferbert A. Corticocortical inhibition in human motor cortex. J Physiol. (1993) 471:501–19. 8120818 10.1113/jphysiol.1993.sp019912 PMC1143973

[40] Rossini PM Burke D Chen R Cohen LG Daskalakis Z Di Iorio R. Non-invasive electrical and magnetic stimulation of the brain, spinal cord, roots and peripheral nerves: Basic principles and procedures for routine clinical and research application. An updated report from an I.F.C.N. Committee. Clin Neurophysiol. (2015) 126:1071–107. 10.1016/j.clinph.2015.02.001 25797650 PMC6350257

[41] Nakamura H Kitagawa H Kawaguchi Y Tsuji H. Intracortical facilitation and inhibition after transcranial magnetic stimulation in conscious humans. J Physiol. (1997) 498:817–23. 9051592 10.1113/jphysiol.1997.sp021905 PMC1159197

[42] Fahn S Williams DT. Psychogenic dystonia. Adv Neurol. (1988) 50:431–55. 3400501

[43] Ridding MC Sheean G Rothwell JC Inzelberg R Kujirai T. Changes in the balance between motor cortical excitation and inhibition in focal, task specific dystonia. J Neurol Neurosurg Psychiatry. (1995) 59:493–8. 8530933 10.1136/jnnp.59.5.493 PMC1073711

[44] Roelofs JJ Teodoro T Edwards MJ. Neuroimaging in functional movement disorders. Curr Neurol Neurosci Rep. (2019) 19:12. 10.1007/s11910-019-0926-y 30747347 PMC6373326

[45] Voon V Cavanna AE Coburn K Sampson S Reeve A LaFrance WC Jr. Functional neuroanatomy and neurophysiology of functional neurological disorders (conversion disorder). J Neuropsychiatry Clin Neurosci. (2016) 28:168–90. 10.1176/appi.neuropsych.14090217 26900733

[46] Edwards MJ Adams RA Brown H Parees I Friston KJ. A bayesian account of 'hysteria'. Brain. (2012) 135(Pt 11):3495–512. 10.1093/brain/aws129 22641838 PMC3501967

[47] Haggard P. Human volition: towards a neuroscience of will. Nat Rev Neurosci. (2008) 9:934–46. 10.1038/nrn2497 19020512

[48] Voon V Gallea C Hattori N Bruno M Ekanayake V Hallett M. The involuntary nature of conversion disorder. Neurology. (2010) 74:223–28. 10.1212/WNL.0b013e3181ca00e9 20083798 PMC2809033

[49] Voon V Brezing C Gallea C Ameli R Roelofs K Hallett1 M. Emotional stimuli and motor conversion disorder. Brain. (2010) 133:1526–36. 10.1093/brain/awq054 20371508 PMC2859149

[50] Voon V Brezing C Gallea C Hallett M. Aberrant supplementary motor complex and limbic activity during motor preparation in motor conversion disorder. Mov Disord. (2011) 26:2396–403. 10.1002/mds.23890 21935985 PMC4162742

[51] Pareés I Saifee TA Kassavetis P Kojovic M Rubio-Agusti I Rothwell JC. Believing is perceiving: mismatch between self-report and actigraphy in psychogenic tremor. Brain. (2012) 135(Pt 1):117–23. 10.1093/brain/awr292. 22075068

[52] Stone J Edwards MJ How “psychogenic” are psychogenic movement disorders? Mov Disord. (2011) 26:1787–88. 10.1002/mds.23882 21761457

[53] Kranick S Ekanayake V Martinez V Ameli R Hallett M Voon V. Psychopathology and psychogenic movement disorders. Mov Disord. (2011) 26:1844–50. 10.1002/mds.23830 21714007 PMC4049464

[54] Hinson VK Cubo E Comella CL Goetz CG Leurgans S. Rating scale for psychogenic movement disorders: scale development and clinimetric testing. Mov Disord. (2005) 20:1592–7. 10.1002/mds.20650 16108025

